# Short telomeres increase the risk of severe COVID-19

**DOI:** 10.18632/aging.104097

**Published:** 2020-10-26

**Authors:** Antoine Froidure, Manon Mahieu, Delphine Hoton, Pierre-François Laterre, Jean Cyr Yombi, Sandra Koenig, Benoit Ghaye, Jean-Philippe Defour, Anabelle Decottignies

**Affiliations:** 1 Department of Pulmonology, Cliniques Universitaires Saint-Luc, Université Catholique de Louvain, Brussels, Belgium; 2 Institut de Recherche Expérimentale et Clinique, Université Catholique de Louvain, Brussels, Belgium; 3 de Duve Institute, Université Catholique de Louvain, Brussels, Belgium; 4 Department of Pathology, Cliniques Universitaires Saint-Luc, Université Catholique de Louvain, Brussels, Belgium; 5 Department of Intensive Care, Cliniques Universitaires Saint-Luc, Université Catholique de Louvain, Brussels, Belgium; 6 Department of Internal Medicine and Infectious Diseases, Cliniques Universitaires Saint-Luc, Université Catholique de Louvain, Brussels, Belgium; 7 Department of Radiology, Cliniques Universitaires Saint-Luc, Université Catholique de Louvain, Brussels, Belgium; 8 Department of Laboratory Hematology, Cliniques Universitaires Saint-Luc, Université Catholique de Louvain, Brussels, Belgium

**Keywords:** COVID-19, telomere length

## Abstract

Telomeres are non-coding DNA sequences that protect chromosome ends and shorten with age. Short telomere length (TL) is associated with chronic diseases and immunosenescence. The main risk factor for mortality of coronavirus disease 2019 (COVID-19) is older age, but outcome is very heterogeneous among individuals of the same age group. Therefore, we hypothesized that TL influences COVID-19-related outcomes.

In a prospective study, we measured TL by Flow-FISH in 70 hospitalized COVID-19 patients and compared TL distribution with our reference cohort of 491 healthy volunteers. We also correlated TL with baseline clinical and biological parameters. We stained autopsy lung tissue from six non-survivor COVID-19 patients to detect senescence-associated β-galactosidase activity, a marker of cellular aging.

We found a significantly higher proportion of patients with short telomeres (<10^th^ percentile) in the COVID-19 patients as compared to the reference cohort (P<0.001). Short telomeres were associated with a higher risk of critical disease, defined as admission to intensive care unit (ICU) or death without ICU. TL was negatively correlated with C-reactive protein and neutrophil-to-lymphocyte ratio. Finally, lung tissue from patients with very short telomeres exhibit signs of senescence in structural and immune cells.

Our results suggest that TL influences the severity of the disease.

## INTRODUCTION

Telomeres are specialized structures that, through the formation of a loop, protect chromosome ends from DNA damage response activation [1]. Telomeres progressively shorten with age, leading to the loss of chromosome end protection and the activation of a p53-dependent DNA damage response that triggers senescence or apoptosis [2, 3]. Short telomeres are associated with a higher risk of aplasia and lung fibrosis, probably linked to early progenitor cell exhaustion [4, 5]. More recently, several studies have demonstrated the impact of shorter telomeres on immune cell function, a phenomenon referred to as immunosenescence. Immunosenescence is an age-dependent process associated with the progressive depletion of naïve T cells and the reduced proliferation ability of T cell that likely impact immune surveillance against persistent viral infections like Cytomegalovirus (CMV) in the elderly [6, 7]. A recent report established that abnormally short telomeres in patients with telomere-related gene mutations are sufficient to drive T cell aging, although additional and still undefined telomere length-independent molecular programs further contribute to immunosenescence in the elderly [8].

Other recent data support a role for telomeres in defense against pathogens: adults with shorter telomeres are more sensitive to experimentally-induced respiratory viral infection [9]. Along the same lines, short leukocyte telomere length (TL) was associated with more severe acute respiratory distress syndrome and worse survival in patients with sepsis [10].

Outcome of the current coronavirus disease 19 (COVID-19) pandemic is highly heterogeneous, ranging from asymptomatic people to patients hospitalized in intensive care units (ICU) with need of mechanical ventilation and eventual fatal outcome due to respiratory failure. To date, the strongest risk factor associated with severe disease and death in COVID-19 is older age [11], with infection fatality rate ranging from eight to 36% in people aged ≥80 years [12]. Yet, studies performed exclusively in younger hospitalized patients –representing the most severely ill patients- reported similar fatality ratios of 8-28%, suggesting that age is not the only factor modulating COVID-19 outcome [12]. Lymphopenia is another risk factor for poor outcome, pointing towards a potential role for telomere modulation in COVID-19 [13, 14].

Based on the above, we hypothesized that shorter TL might be linked to poorer outcome in COVID-19 and addressed this hypothesis in a prospective cohort of hospitalized patients.

## RESULTS

### Study population

We prospectively recruited 70 patients hospitalized in COVID-19 dedicated units between April 7^th^ and May 27^th^, 2020, during the main wave of COVID-19 in Belgium. The clinical characteristics of our patients are provided in Table 1. Our cohort included 48 men (68.6%). Median age was 63 years-old (range 27-96). Fifty-three (75.7%) patients had at least one chronic disease including hypertension (22, 31.4%), previously documented hypercholesterolemia (20, 28.6%), diabetes (13, 18.6%), obesity defined as a BMI>30 kg/m² (9, 12.9%) or ischemic cardiovascular disease (8, 11.4%). Twenty-five patients (35.7%) were current or ex-smokers. At the day of admission in the hospital (baseline), no patient had received any COVID-19-related treatment. During their hospitalization, a majority of subjects received hydroxychloroquine (59, 84.3%), as this drug was at that time recommended for hospitalized patients in Belgium. Eleven patients (15.7%) experienced a thrombotic event requiring therapeutic anticoagulation (five deep venous thrombosis, three arterial thrombosis, two ischemic strokes and one pulmonary embolism). During hospitalization, median peak oxygen flow was 10 liter/minute administered through a mask, which corresponds to a fraction of inspired oxygen of about 0.9. Fourteen patients (20%) beneficiated from continuous positive airway pressure (CPAP).

**Table 1:** Clinical characteristics of the TELECOVID cohort (all values are median and range, unless specified).

**Baseline clinical features**	
Age (years)	63 (27-96)
Sex ratio (M/F)	48/22
Ethnicity (N)	49 Caucasians, 11 Northern Africans, 5 Asians, 4 Africans, 1 Southern American
Current or ex-smokers (N, %)	25 (35.7%)
Hypertension	22 (31.4%)
Known hypercholesterolaemia (N, %)	20 (28.6%)
Diabetes	13 (18.6%)
Obesity (BMI>30 kg/m²) (N, %)	9 (12.9%)
Ischemic cardiovascular disease	8 (11.4%)
Symptoms duration prior to admission (days)	7 (1-15)
Disease extend on HRCT (%)*	20.61 (0.47-68.78)
**Baseline biological features**	
CRP (mg/L)	95.95 (1.3-353.2)
LDH (IU/L)	372 (162-1855)
ASAT/GOT (U/L)	41 (8-242)
PaO_2_with fraction of inspired oxygen 0.21 (ambient air, mmHg)	64.5 (26-134)
Blood lymphocytes (x10³/μL)	0.68 (0.17-2.13)
Blood neutrophils (x10³/μL)	4.99 (0.48-15.91)
Neutrophils/lymphocytes ratio	6.03 (0.86-50.57)
Eosinophils (x10³/μL)	0 (0-0.18)
**COVID-19 management characteristics**	
Hospitalisation duration (days)†	19.5 (4-102)
Specific treatment (N, %), including - Hydroxychloroquine (N, %) - Systemic corticosteroids (N, %) - Others (N, %)	59 (84.3%) - 59 (84.3%) - 5 (7.1%) - 3 (4.2%)‡
Admission in ICU (N, %)	33 (47.1%)
Days in ICU‡	23 (2-70)
Death (N, %)	18 (25.7%)

*: absence of baseline HRCT for 8 patients.

†: 2 missing data (patients still hospitalised).

‡: 1 patients received an anti-IL-6 monoclonal antibody, 2 patients received azithromycin.

Thirty-three patients (47.1%) were admitted in intensive care, of whom 30 patients required mechanical ventilation (42.8%). Nine patients (12.9%) benefited from extra-corporeal membrane oxygenation (ECMO). Eighteen patients (25.7%) died from COVID-19.

### Increased frequency of short telomere individuals in hospitalized COVID-19 patients

**Figure 1:**
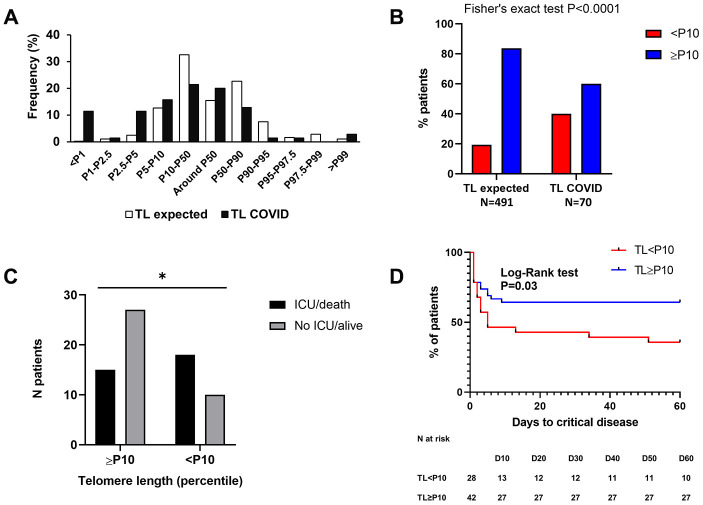
**High proportion of COVID-19 patients with short telomeres and link with outcome.** (**A**) As compared to the reference population (white bars), we found an enrichment of COVID-19 patients with short telomeres (black bars). (**B**) There is a statistically significant association between short telomeres (<P10) and COVID-19 (Chi-square test, P<0.0001). (**C**) There is a statistically significant association between short telomeres (<P10) and unfavorable outcome (admission in ICU and/or death) in COVID-19 hospitalized patients (Chi-square test, P=0.02). (**D**) Kaplan-Meyer curve showing the time to admission in ICU and/or death without in patients with TL <P10 or ≥P10.

We measured TL in leucocytes using the Flow-FISH technique [15]. Figure 1A shows the distribution of individuals within the indicated percentile ranges of TL. When compared to the reference cohort, we found a clear enrichment of patients with telomeres <P10 (N=28, 40.0%, Figure 1B (p<0.0001, Chi-Square test)), indicating that hospitalized COVID-19 patients display significantly shorter telomeres than the reference population.

### Short telomeres are associated with a higher risk of unfavorable COVID-19 outcome and correlate with prognostic biological factors

We segregated our population based on short (<10^th^ percentile, P10) or normal (≥P10) telomere length (Table 2). Twenty-eight (40%) patients had a TL<P10 and 42 had a TL≥P10.

**Table 2:** Comparison of key clinical and biological features between patients with telomere length below and equal or above the 10^th^ percentile.

	***TL < 10^th^ percentile (N=28)***	***TL ≥ 10^th^ percentile (N=42)***	***P-value (Mann-Whitney U test)***
**Clinical data and outcome**			
Age (years, range)	64 (47-85)	62.5 (27-96)	0.21^μ^
Sex ratio (M/F)	23/5	25/17	0.07*
HRCT extend (%, range)	23.80 (2.28-68.78)	18.57 (0.47-68.39)	0.41
Hospitalization duration (days, range) †	24 (6-96)	16 (4-74)	0.10
Death	10 (35.7%)	8 (19.0%)	0.16
Admission in ICU and/or death	18 (64.3%)	15 (35.7%)	0.02*
**Biological features**			
NLR (median, range)	7.65 (3.08-50.57)	5.36 (1.23-25.6)	**0.01**
Blood lymphocytes at admission (x10³/μL, median, range)	0.68 (0.17-2.01)	0.74 (0.19-2.13)	0.69
Blood neutrophils at admission (x10³/μL, median, range)	5.17 (1.98-12.15)	4.36 (0.48-15.91)	0.20
CRP at admission (mg/L, median, range)	120 (8.6-353.2)	71.15 (1.3-330.8)	0.11

μ: Student T-test

*: Fisher’s exact test

$: Chi-Square test

†: 2 missing data (patients still hospitalised)

We found that TL<P10 was significantly associated with a greater risk to develop a critical disease, namely being admitted in ICU or death without ICU (composite endpoint), OR 3.24 (CI 1.21-8.55, P=0.02, Chi-square test Figure 1C). We also built a Kaplan-Meyer curve of our populations, showing time elapsed prior to critical disease. There was a significant difference between patients with TL<P10 and patients with TL ≥P10 as shown in Figure 1D. There was also a trend for higher mortality in the TL<P10 group as compared to patients with TL≥P10, but this was not statistically significant (32.1% *VS* 21.4%, P=0.16). Furthermore, TL (in percentiles) negatively correlated with hospitalization duration (Spearman r=-0.248, P=0.041). Importantly, there was no significant difference of age between patients with telomeres <P10 and ≥P10 (Median age 64 *VS* 62.5, P=0.21, Table 2). As shown in Table 2, neutrophil-to-lymphocyte ratio (NLR) was significantly higher in patients with TL below 10, although neutrophil counts were similar. So, the significant difference in NLR was mainly driven by lymphopenia.

We then studied the correlation between TL (expressed in percentile) and biological factors previously associated with poorer COVID-19 outcome, namely C-reactive protein level (CRP), lymphocyte count, NLR and eosinophils. We found a moderate but statistically significant correlation between TL and CRP (Spearman r = -0.259, P=0.03) and NLR (Spearman r = -0.268, P=0.025), meaning that the lower the TL, the higher the CRP and NLR. There was a trend for a significant correlation between TL and lymphocyte count (Spearman r = 0.197, P=0.11), suggesting that the longer the telomeres, the higher the blood lymphocytes at admission.

### TL is not correlated with high resolution computed tomography (HRCT) extend of COVID-19-related lesions

Out of the 70 patients, 62 (88.6%) underwent a HRCT on the day of admission. We compared the extent of lesion on HRCT (percentage opacity score) at admission between patients with TL<P10 and ≥P10 (median, range) and did not find any difference between both groups (23.8%, 2.3-68.8 *vs.* 18.6%, 0.5-68.4, P=0.41, Mann-Whitney *U* test).

### Increased cellular senescence in lungs from COVID-19 patients with very short telomeres

**Figure 2:**
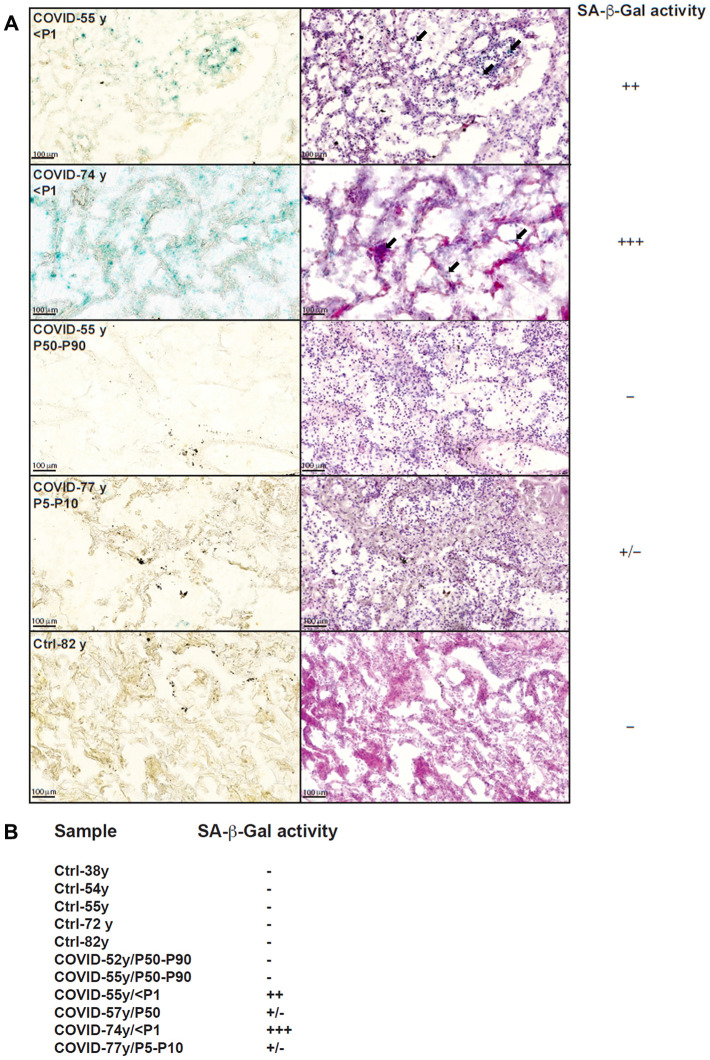
**SA-β-Gal staining of lung slices from COVID-19 and control patients.** (**A**) SA-β-Gal staining (left) and HE staining (right) of four COVID-19 lungs and one control lung. We only found signs of senescence (blue staining) in lungs from individuals with very short telomeres (<P1), both in structural and immune cells (black arrows). Conversely, staining was weak for a patient with short telomeres and absent for patients with normal TL and in control tissue. (**B**) overview of detected SA-β-Gal activity within analyzed samples.

To evaluate whether short telomeres of COVID-19 patients may be associated with immune cell senescence, we evaluated SA-β-gal activity in lungs from non-survivor patients. Our results revealed high levels of SA-β-gal activity in both immune and structural lung cells from two COVID-19 patients of 55 and 74 years old with TL below the first percentile (P1) (Figure 2A). We did not detect any SA-β-gal activity in the lung of another 55 years old deceased COVID-19 patient with telomeres lying in the P50-P90 range (Figure 2A). In the remaining lungs from deceased patients with TL >P1, low to moderate levels of SA-β-gal activity were detected (Figure 2A, 2B). As previously demonstrated [16], negative controls obtained from healthy lung tissues of donors aged 38-82 years old did not reveal any SA-β-gal activity (Figure 2A, 2B).

## DISCUSSION

Our study in a cohort of 70 hospitalized COVID-19 patients revealed an enrichment for individuals with short telomeres in comparison with the reference population. Furthermore, short telomeres (<P10) were associated with a higher risk of admission in ICU and/or death independently of age. TL was significantly correlated to CRP and neutrophil-to-lymphocyte ratio, both factors previously associated with poorer COVID-19 outcome [13]. Finally, we found evidence of senescence in both structural and immune cells in the lungs of very short telomere (<P1) patients who died from COVID-19. Altogether, our findings support the hypothesis that TL modulates COVID-19 outcome and may partially explain the high variability of COVID-19 outcome among individuals of similar age. The mild correlations between TL and biological factors in our cohort suggest that TL is probably one feature, among others, such as variants in immune-related genes, type 2 angiotensin convertase enzyme [17] or variations in the ABO blood-group system, that underpin impaired inflammatory response towards coronavirus 2. On the contrary, we did not find any link between TL and extent of lesion on HRCT, although the latter is a major determinant of COVID-19-related mortality. This is probably because ground glass opacities and condensations, key radiological features on HRCT [18], do not only reflect the presence of inflammation but also edema, bronchiolitis and ventilation defects.

The progressive shortening of lymphocyte telomeres with aging has been proposed to contribute to the age-associated defects of immunity and the higher risk of long-term infection with CMV [6] and mortality [19]. Conversely, healthy elderly exhibit longer telomeres than their peers do [20, 21]. Beyond COVID-19 and other viral diseases, differences in TL and telomere homeostasis may underlie impaired immune response towards various pathogens, as illustrated by the recent demonstration that sepsis outcome was also linked to TL [10].

We [22] and others previously showed that prematurely aged skin fibroblasts from patients with abnormally short telomeres display increased SA-β-gal activity, a marker first described in senescent skin fibroblasts and keratinocytes. Here, we only found signs of senescence in the lungs of non-survivors with very short telomeres (<P1) and not in either patients with normal TL or aged controls, which further illustrates that the observed senescence in the two COVID-19 patients with TL <P1 is linked to the presence of very short telomeres, and not to age. Of note, we cannot exclude that lung senescence was a pre-existing condition in these patients with very short telomeres, but neither of these patients had a preexisting lung condition. So, based on our observations, it is very unlikely that lung senescence constitutes a driver for COVID-19, but it may have been an aggravating factor in those two patients.

Short telomeres also possibly impact COVID-19 outcome through the modulation of cytokine production, as illustrated by our finding that TL correlated with NLR, which constitutes a prognostic factor in multiple diseases [23], and CRP. Along this line, the increased transcription of short telomeres into TERRA non-coding RNAs was reported to promote IL-6 and TNF-α secretion [24, 25]. Short telomeres have also been associated with an increased transcription of *ISG15* [26], a gene related to Type 1 interferon signaling. Hence, short telomeres may also possibly contribute to the harmful cytokine storm of COVID-19 patients.

As a limitation, our cohort was composed of severely to critically ill patients, with about a half of them being admitted to ICU and a fatality rate of 25.6%. Therefore, whether our result may apply to the overall population of COVID-19 patients still needs to be confirmed.

In conclusion, we uncovered a link between telomere length and COVID-19 outcome, with a potential impact of TL on biological parameters. Altogether, our study paves the way for further investigations on a potential interest of TL as a prognostic factor of COVID-19. Whether TL may influence long-term outcome like lung fibrosis is also unknown at this point.

## MATERIALS AND METHODS

### Study design

The TElomere LEngth in COronaVIrus Disease 2019 (TELECOVID) trial is a single-center prospective trial. We prospectively included patients admitted in our dedicated COVID-19 units that fulfilled the following criteria: evidence of COVID-19 defined as the combination of a positive PCR on nasopharyngeal swab and lung infiltrates on high resolution computed tomography (HRCT) or chest X-ray at admission. All patients underwent blood sampling for TL measurement on peripheral blood granulocytes. We collected clinical, biological and radiological data on the day of admission in the hospital. We quantified the extension of lesions on admission HRCT with the CT Pneumonia Analysis software (Siemens Helthineers, Forchheim, Germany) and obtained a percentage opacity score, defined as the percentage of predicted volume of abnormalities compared to the total lung volume.

TL measurements obtained in COVID-19 patients were compared to those of a reference cohort of 491 healthy volunteers (from 0 to 99 years) obtained in the Cliniques Universitaires Saint-Luc (ISO15189).

We have conducted this cohort study in accordance with STROBE statement (https://www.strobe-statement.org/index.php?id=strobe-home).

### Study objectives

Our primary objectives were to measure telomere length in hospitalized COVID-19 patients and compare it to nomograms from our reference population and to study the potential association between short telomeres (below tenth percentile -P10) and unfavorable COVID-19 outcome, defined as admission in ICU and/or death without ICU (critical disease, according to the definition of NIH, covid19treatmentguidelines.nih.gov/overview/management-of-covid-19/). Our secondary objectives were to correlate TL to baseline (at the day of admission in hospital) biological and radiological features. Finally, we sought to detect signs of senescence in lung samples from deceased COVID-19 patients.

### Blood sample processing and telomere length (TL) measurement

TL was measured by Flow-FISH, currently considered as the gold standard technique [27]. The severe lymphopenia of most COVID-19 patients, together with the possible acute infection-driven shortening of telomeres in T lymphocytes, support the measurement of TL in the granulocytes rather than the lymphocytes. To confirm that granulocyte TL properly reflects lymphocyte TL, we compared their respective percentile ranges for individuals of the cohort. We found a good consistency between the two cell populations as, for nearly 60% of the individuals of the cohort, the percentile range was the same while, for 33% of them, only one category difference was observed (Supplementary Figure 1). Hence, for the large majority of individuals, granulocyte TL properly reflects lymphocyte TL.

All TL measurements were performed in duplicate in the Cliniques Universitaires Saint-Luc through Flow-FISH as described previously [15]. After red blood cell lysis with ammonium chloride as described in [15], white blood cells were frozen at -80° C until analysis. Aliquots of calf thymus were mixed to each sample for internal control. Telomeres were stained with FITC-labelled (CCCTAA)_3_ PNA probe (Panagene) and fluorescence was measured with a Navios EX flow cytometer (Beckman Coulter).

### Senescence-associated-β-galactosidase activity in lung tissues

We had access to autopsy lung tissue from COVID-19 deceased patients and control lung tissue from our local biobank (IREC-PNEU). We slightly adapted the protocol previously described to perform senescence-associated β-galactosidase (SA-β-Gal) [28]. Briefly, after fixation with 0.2% formaldehyde for 10 min, 16 μm-thick sections of snap-frozen lung tissues were rinsed with PBS before overnight incubation at 37° C in staining solution at pH 5.6. After scanning, slides were incubated overnight in PBS 1x at RT, rinsed with ddH_2_0 and stained first with hematoxylin and then with eosin. After a new rinsing with water, tissues were dehydrated through a series of ethanol baths, from 30% to 100%, before incubation in isopropanol and then xylene and, finally, mounting.

### Statistics

We used Chi-Square and Fisher’s exact test for associations and Spearman *R* test for correlation analysis. We used Student *t* test and Mann-Whitney *U* test for single comparisons when appropriate. We performed a Kaplan-Meyer curve and used Log-Rank test for comparing populations. All statistics were performed on SPSS 25 software (IBM, Armonk, NY, USA) and GraphPad Prism 8.4.1 (Graphpad Software, San Diego, CA, USA). A P-value under 0.05 was considered significant.

### Ethical considerations

All patients provided informed consent prior to inclusion. This study was approved by our internal review board (Comité d’éthique hospitalo-facultaire), approval number 2020/06AVR/200.

## Supplementary Material

TELECOVID Investigators

Supplementary Figure 1
