# Supplementary material for: Short telomeres increase the risk of severe COVID-19
Source: Aging (Albany NY). 2020 Oct 26;12(20):19911–22. doi: 10.18632/aging.104097 (PMC7655194; doi:10.18632/aging.104097)
Supplement: TELECOVID Investigators [file aging-12-104097-s001..pdf]

SUPPLEMENTARY MATERIALS

TELECOVID Investigators

Frank Aboubakar[1,2], Christophe Beauloye[9,2], Leila Belkhir[6,2], Emmanuel Coche[7,2], Ludovic Gérard[5,2], Sophie Gohy[1,2], Luc-Marie Jacquet[5,2], Giuseppe Liistro[1,2], Amin Mahsouli[7,2], Benny Gimbada Mwenge[1,2], Thierry Pieters[1,2], Charles Pilette[1,2], Olivier Vancaeneghem[5,2]

[1]Department of Pulmonology, Cliniques universitaires Saint-Luc, Université Catholique de Louvain, Brussels, Belgium
[2]Institut de Recherche Expérimentale et Clinique, Université Catholique de Louvain, Brussels, Belgium
[3]de Duve Institute, Université catholique de Louvain, Brussels, Belgium
[4]Department of Pathology, Cliniques Universitaires Saint-Luc, Université Catholique de Louvain, Brussels, Belgium
[5]Department of Intensive Care, Cliniques Universitaires Saint-Luc, Université Catholique de Louvain, Brussels, Belgium
[6]Department of Internal Medicine and Infectious Diseases, Cliniques Universitaires Saint-Luc, Université Catholique de Louvain, Brussels, Belgium
[7]Department of Radiology, Cliniques Universitaires Saint-Luc, Université Catholique de Louvain, Brussels, Belgium
[8]Department of Laboratory Hematology, Cliniques Universitaires Saint-Luc, Université Catholique de Louvain, Brussels, Belgium
[9]Department of Cardiology, Cliniques Universitaires Saint-Luc, Université Catholique de Louvain, Brussels, Belgium
