# Supplementary material for: Short telomeres increase the risk of severe COVID-19
Source: Aging (Albany NY). 2020 Oct 26;12(20):19911–22. doi: 10.18632/aging.104097 (PMC7655194; doi:10.18632/aging.104097)
Supplement: Supplementary Figure 1 [file aging-12-104097-s002..pdf]

SUPPLEMENTARY FIGURE

A

Telomere length categories										
<P1	P1-P2.5	P2.5-P5	P5-P10	P10-P50	Around P50	P50-P90	P90-P95	P95-P97.5	P97.5-P99	>P99

B

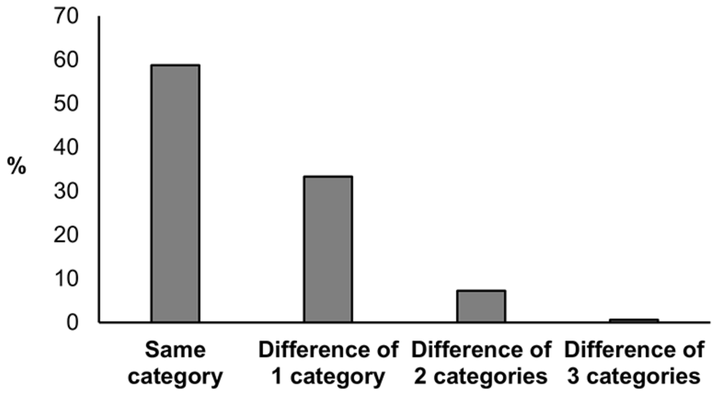

Supplementary Figure 1. Telomere length is consistent in lymphocytes and granulocytes. TL is categorized as described in (**A**). When we compared TL in lymphocytes and granulocytes of patients from our cohort, more than 90% of patients had no (58.7%) or only one (33.3%) category difference in TL (**B**).
